# Mega riverbed-patterns: linear and weakly nonlinear perspectives

**DOI:** 10.1098/rspa.2021.0331

**Published:** 2021-08

**Authors:** Sk Zeeshan Ali, Subhasish Dey, Rajesh K. Mahato

**Affiliations:** ^1^ Department of Civil Engineering, Indian Institute of Technology Hyderabad, Telangana 502284, India; ^2^ Department of Civil Engineering, Indian Institute of Technology Kharagpur, West Bengal 721302, India

**Keywords:** river dynamics, instability, sediment transport

## Abstract

In this paper, we explore the mega riverbed-patterns, whose longitudinal and vertical length dimensions scale with a few channel widths and the flow depth, respectively. We perform the stability analyses from both linear and weakly nonlinear perspectives by considering a steady-uniform flow in an erodible straight channel comprising a uniform sediment size. The mathematical framework stands on the dynamic coupling between the depth-averaged flow model and the particle transport model including both bedload and suspended load via the Exner equation, which drives the pattern formation. From the linear perspective, we employ the standard linearization technique by superimposing the periodic perturbations on the undisturbed system to find the dispersion relationship. From the weakly nonlinear perspective, we apply the centre–manifold-projection technique, where the fast dynamics of stable modes is projected on the slow dynamics of weakly unstable modes to obtain the Stuart–Landau equation for the amplitude dynamics. We examine the marginal stability, growth rate and amplitude of patterns for a given quintet formed by the channel aspect ratio, wavenumber of patterns, shear Reynolds number, Shields number and relative roughness number. This study highlights the sensitivity of pattern formation to the key parameters and shows how the classical results can be reconstructed on the parameter space.

## 1. Introduction

Rhythmic riverbed-patterns fascinate geophysicists. They are formed when a turbulent flow shears an erodible channel-bed by entraining sediment particles into the flow. They remain a major part in the history of river evolution [1,2]. Mega riverbed-patterns are the configurations, whose longitudinal and vertical length scales are of the order of a few channel widths and flow depth, respectively [3]. For example, the classic architecture of large erosion and depositional patterns (e.g. alternate bars) comprises a longitudinal wavelength up to tenfold the river width, a transverse wavelength of the order of the river width and a finite amplitude that scales with the flow depth. The mega riverbed-patterns are classified either from a mechanistic perspective (e.g. free and forced bars, steady and migrating bars, and central and braided bars) or from a topological perspective (e.g. longitudinal bars, transverse bars and diagonal bars) [4].

Mega riverbed-patterns appear in several engineering problems. The riverbed erosion favours the local scour at structures (e.g. bridge piers), whereas the riverbed deposition reduces the width of the navigation channel, enhancing the flood risk [5]. Further, the mega riverbed-patterns help in the growth of riparian plants creating aquatic habitats [6]. In particular, the formation of incipient alternate bars explains the possible origin of the onset of meandering of rivers [7], while the central and braided bars cause the braiding of rivers [4]. River bars may develop as a single or periodic repetitions of deep and shallow reaches. The interplay between the carrier fluid and the erodible channel-bed under certain physical conditions gives rise to periodic patterns [8]. The patterns that are free from any external forcing are called the free bars [9], e.g. alternate, central and braided bars. Alternate bars develop at the left and right channel-banks, while central bars arise at the middle of the channel. In addition, braided bars appear to be several depositional features at any cross section of the channel. The central and braided bars are usually found in wide channels [9].

In addition to a plethora of studies carried out through laboratory experiments [10–18], field measurements [19–23] and numerical simulations [24,25], mega riverbed-patterns were largely analysed theoretically [3,9,26–35]. In the theoretical framework, the riverbed-patterns are thought of as double-harmonic waves that develop as a consequence of the so-called instability phenomenon, which arises as a response of the fluid–riverbed interface to the flow and the particle transport [8]. The theoretical studies on mega riverbed-patterns mainly considered the depth-averaged assumption for the momentum and continuity equations of flow, although this assumption was relaxed elsewhere [9]. In addition, the sediment continuity equation, e.g. Exner equation, is applied to find the dynamics of bed evolution. The theoretical studies are founded on two major perspectives, such as linear and nonlinear perspectives.

The linear perspective simplifies the dynamics of patterns by applying the standard linearization technique. In this technique, the perturbation of a generic variable is considered to be much smaller than its undisturbed value. Moreover, the perturbations are assumed to follow harmonic functions in accordance with the Fourier theorem. For a given flow and particle transport conditions, the mega riverbed-patterns are expected to form above a threshold aspect ratio (e.g. channel half-width to flow depth ratio) [3]. The linear perspective puts together all the key quantities, e.g. growth rate, wavelength and migration speed of patterns, in a single framework. On the other hand, the nonlinear perspective predicts the amplitude of patterns. This perspective includes both weakly nonlinear [3,26,32] and fully nonlinear [36] theories. The latter is also known as an axisymmetric approach for forced patterns [4].

The role of particle transport towards the dynamics of mega riverbed-patterns remains a crucial aspect. Although most of the theoretical studies have analysed the pattern formation in bedload-dominated channels [3,28,29,31], some studies have taken into account the effects of sediment suspension in addition to the bedload transport [9,30,32]. Sediment suspension was incorporated into the theoretical framework either by means of a fully three-dimensional approach [9] or through an extension of the depth-averaged formulation [37] to two dimensions [30]. The effects of sediment suspension were found to lower the threshold aspect ratio, enhancing the unstable domain of patterns significantly. In bedload-dominated channels, the nature of mega riverbed-patterns was found to be convective [29,31], as any tiny local disturbance is convected downstream without disturbing the flow domain over a long duration [30]. However, the convective nature of patterns does not appear to have changed even in the presence of sediment suspension [30]. The mega riverbed-patterns have been also analysed theoretically considering the effects of unsteadiness [28,31], vegetation [38,39] and sediment heterogeneity [27]. The sediment heterogeneity causing the selective transport of particles was found to dampen the wavelength, growth rate and migration speed of patterns [27].

The weakly nonlinear analysis for bedload-dominated channels was carried out by using the multiple-scale technique [3] and the centre-manifold-projection technique [39], while that for combined bedload and suspended load transport was performed by using the centre-manifold-projection technique [32], extending the previous linear analysis [30]. In the weakly nonlinear analysis, the amplitudes of patterns for both plane and dune-covered beds were predicted [32]. It was found that the presence of dunes is to enhance the equilibrium amplitude of patterns.

Despite magnificent advances in studying the mega riverbed-patterns theoretically [4,8], little is known about how the patterns are sensitive to a class of parameters pertinent to flow and particle transport. Although a recent study has clearly shown that the inclusion of sediment suspension changes the stability and amplitude of patterns significantly [32], more insights are to be gained regarding how and why these changes evolve with the characteristic parameters. Moreover, the behaviours of the growth rate and amplitude of patterns in transitional and rough flow regimes need to be examined.

In this paper, we analyse the mega riverbed-patterns, being free from an external forcing, from both linear and weakly nonlinear perspectives. Employing the depth-averaged formulations for the flow and particle transport, we follow the standard mathematical techniques to analyse the pattern formation. From the linear perspective, we apply the standard linearization technique, whereas from the weakly nonlinear perspective, we apply the centre-manifold-projection technique [32]. The main objective of the linear stability analysis is to explore how the mega riverbed-patterns are sensitive to the characteristic parameters, e.g. channel aspect ratio, shear Reynolds number, Shields number and relative roughness number. In addition, the primary objective of the weakly nonlinear stability analysis is to explore the sensitivity of the finite amplitude of patterns to the key parameters.

The paper is organized as follows. In §2, the theoretical analysis of flow is presented. The theoretical analysis of particle transport is described in §3. The stability analyses from the linear and weakly nonlinear perspectives are furnished in §4. The computational results are discussed in §5. Finally, the conclusion is drawn in §6.

## 2. Theoretical analysis of flow

**Figure 1.**
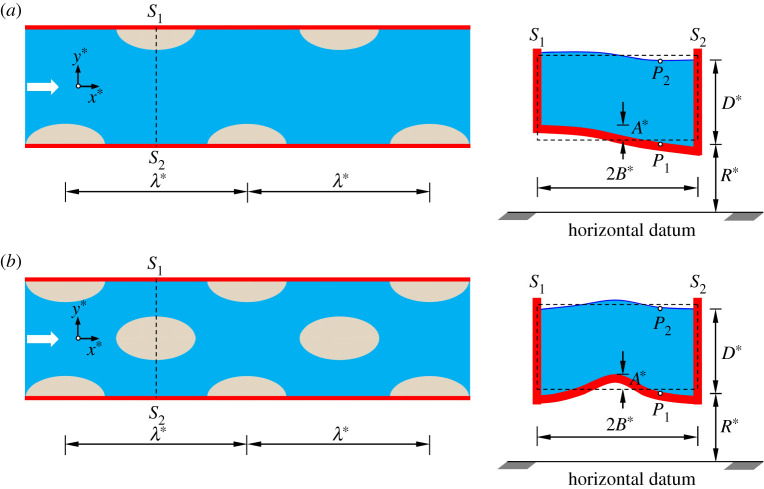
Conceptual sketch of mega riverbed-patterns: (*a*) alternate bars and (*b*) central bars. (Online version in colour.)

We consider mega riverbed-patterns, having a wavelength $\lambda^{*}$ and a finite amplitude $A^{*}$, driven by a free-surface turbulent flow in an infinitely long straight channel of width 2$B^{*}$ (figure 1*a*,*b*). Henceforth, a star superscript is used to represent a dimensional variable. We employ a Cartesian coordinate system ($x^{*},y^{*},z^{*}$), where $x^{*}$, $y^{*}$ and $z^{*}$ are the longitudinal, transverse and vertical distances, respectively. The origin of the coordinate system lies on the centreline of the channel. Two well-recognized patterns are highlighted in figure 1. In figure 1*a*, the alternate bars are shown, where the channel embraces a single row of bars. On the other hand, in figure 1*b*, the central bars are shown, covering two parallel rows of alternate bars. The presence of bars causes channel-bed oscillations in both longitudinal and transverse directions. For alternate bars (figure 1*a*), a typical section $S_{1}$–$S_{2}$ across the channel shows the channel-bed erosion and deposition near the right and left channel-banks, respectively. In addition, for central bars (figure 1*b*), the cross-sectional view shows the channel-bed erosion and deposition near the channel-banks and at the channel-bed centreline, respectively. At a given time $t^{*}$, $D^{*}(x^{*},y^{*},t^{*})$ is the local flow depth, which is the vertical distance between two characteristic points $P_{1}$ and $P_{2}$ at a given transverse distance (figure 1*a*,*b*). In addition, $z^{*}=R^{*}(x^{*},y^{*},t^{*})$ denotes the elevation of the channel-bed from a fixed horizontal datum. Note that in figure 1, the dashed rectangular section represents the undisturbed cross section of flow.

In the theoretical analysis, we employ the quasi-steady and the depth-averaged assumptions. The former is appropriate here, as the time scale of the morphological patterns is quite larger than that of the flow. In addition, the latter assumption emerges from the fact that the wavelength of the mega riverbed-patterns scales with a few channel widths [3]. Now, we introduce the pertinent variables in dimensionless form as follows:

2.1 $$(x,y)=\frac{\left(x^{*},y^{*}\right)}{B^{*}},\;t=\frac{t^{*}U_{0}^{*}}{B^{*}},\;(R,D)=\frac{\left(R^{*},D^{*}\right)}{D_{0}^{*}},\;U=\frac{U^{*}}{U_{0}^{*}},\;T=\frac{T^{*}}{\rho_{f}U_{0}^{*2}},$$

where $D_{0}^{*}$ and $U_{0}^{*}$ are the undisturbed flow depth and flow velocity (i.e. the flow depth and flow velocity corresponding to the uniform flow), respectively, $U^{*}=(U_{x}^{*},U_{y}^{*})$ is the depth-averaged velocity vector, $T^{*}=(T_{x}^{*},T_{y}^{*})$ is the bed shear stress vector and $\rho_{f}$ is the mass density of fluid. Moreover, we introduce the channel aspect ratio β and the undisturbed flow Froude number Fr as

2.2 $$\beta =\frac{B^{*}}{D_{0}^{*}}\;\text{and}\;Fr=\frac{U_{0}^{*}}{{\left(gD_{0}^{*}\right)}^{1/2}},$$

where g is the acceleration due to gravity.

The depth-averaged momentum and continuity equations of flow are expressed as

2.3 $$\begin{array}{rl} & U_{x}\frac{\partial U_{x}}{\partial x}+U_{y}\frac{\partial U_{x}}{\partial y}+\frac{1}{Fr^{2}}\frac{\partial }{\partial x}(D+R)+\beta \frac{T_{x}}{D}=0, \end{array}$$

2.4 $$\begin{array}{rl} & U_{x}\frac{\partial U_{y}}{\partial x}+U_{y}\frac{\partial U_{y}}{\partial y}+\frac{1}{Fr^{2}}\frac{\partial }{\partial y}(D+R)+\beta \frac{T_{y}}{D}=0 \end{array}$$

2.5 $$\begin{array}{rl} \text{and}\; & \frac{\partial }{\partial x}(DU_{x})+\frac{\partial }{\partial y}(DU_{y})=0. \end{array}$$

In order to close the hydrodynamic equations, an estimation of the bed shear stress vector is essential. The bed shear stress vector is expressed herein as a function of dynamic pressure. Hence, we write

2.6 $$T=(T_{x},T_{y})=C_{f}{\left(U_{x}^{2}+U_{y}^{2}\right)}^{1/2}(U_{x},U_{y}),$$

where $C_{f}$ is the conductance factor. It can be linked with the Darcy–Weisbach friction factor f as $f=8C_{f}$. The $C_{f}$ and f can be obtained from the Colebrook–White equation as follows [40]:

2.7 $$\frac{1}{{\left(8C_{f}\right)}^{1/2}}=\frac{1}{f^{1/2}}=-0.86\ln\left[{\left(\frac{k_{s}^{*}}{a_{r}D^{*}}\right)}^{1.1}+\frac{1}{f^{1/2}}\cdot \frac{a_{s}}{Re}\right]\;\text{with}\;Re=\frac{4U_{x}^{*}D^{*}}{\upsilon },$$

where $k_{s}^{*}$ is the bed roughness height (= $\alpha d^{*}$), α is a coefficient (=2.5) [41], $d^{*}$ is the particle size, $a_{r}$ and $a_{s}$ are the constants (=14.8 and 2.51, respectively), Re is the flow Reynolds number and υ is the coefficient of kinematic viscosity of fluid. The above equation provides an accurate estimation of the friction factor over a wide range of flow regimes. The shear Reynolds number $R_{*}$ is commonly used to demarcate the flow regimes [42]. The $R_{*}$ is defined as

2.8 $$R_{*}=\frac{u_{f}^{*}k_{s}^{*}}{\upsilon }\;\text{with}\;u_{f}^{*}={\left(\frac{|T^{*}|}{\rho_{f}}\right)}^{1/2},$$

where $u_{f}^{*}$ is the shear velocity.

## 3. Theoretical analysis of particle transport

The erodible channel-bed comprises uniform cohesionless sediment particles. The flow over the channel-bed exerts hydrodynamic force on the particles. The particles are entrained into the flow when the dimensionless fluid-induced bed shear stress crosses its threshold value $\Theta_{c}$ [43]. Both the modes of particle transport as bedload and suspended load transport are considered herein. The bed shear stress is quantified by introducing the Shields number Θ as follows:

3.1 $$\Theta =\frac{|T^{*}|}{\rho_{f}(s-1)gd^{*}},$$

where s is the relative density ($=\rho_{p}/\rho_{f}$) and $\rho_{p}$ is the mass density of particles. It is pertinent to mention that the suspended load transport prevails when the Shields number Θ exceeds its threshold value $\Theta_{s}$ to bring the particles into the suspension. Hence, for $\Theta_{c}<\Theta <\Theta_{s}$, the bedload remains the dominant mode of particle transport, while for $\Theta >\Theta_{s}$, both the bedload and suspended load transport take place. For the estimation of $\Theta_{s}$, we use van Rijn’s [44] empirical formulae as

3.2 $$\Theta_{s}(1<D_{*}\leq 10)=\frac{16}{D_{*}^{2}}\cdot \frac{w_{s}^{*2}}{(s-1)gd^{*}},\;\Theta_{s}(D_{*}>10)=0.16\frac{w_{s}^{*2}}{(s-1)gd^{*}}.$$

In the above, $D_{*}=d{\left[(s-1)g/\upsilon^{2}\right]}^{1/3}$, called the particle parameter and $w_{s}^{*}$ is the settling velocity of particles. To find $w_{s}^{*}$, we use Jiménez & Madsen’s [45] empirical formula as

3.3 $$\frac{w_{s}^{*}}{{\left[(s-1)gd^{*}\right]}^{1/2}}={\left(0.954+\frac{20.48}{D_{*}^{3/2}}\right)}^{-1}.$$

The shear Reynolds number $R_{*}$ can be linked with Θ and $D_{*}$ as

3.4 $$R_{*}=\alpha {\left(\Theta D_{*}^{3}\right)}^{1/2}.$$

Further, with the introduction of relative roughness number $d_{r}(=d^{*}/D_{0}^{*})$, the flow Froude number Fr can be expressed as

3.5 $$Fr={\left[\frac{(s-1)d_{r}\Theta }{C_{f}}\right]}^{1/2}.$$

The dynamics of bed evolution is governed by the Exner equation. It is expressed as

3.6 $$\frac{\partial R}{\partial t}+Q_{0}\left(\frac{\partial \Phi_{bx}}{\partial x}+\frac{\partial \Phi_{by}}{\partial y}\right)+\frac{\partial \Phi_{sx}}{\partial x}+\frac{\partial \Phi_{sy}}{\partial y}=0,$$

where

3.7 $$Q_{0}=\frac{1}{1-\rho_{0}}\cdot \frac{{\left[(s-1)gd^{*3}\right]}^{1/2}}{U_{0}^{*}D_{0}^{*}},\;\Phi_{b}=\frac{Q_{b}^{*}}{{\left[(s-1)gd^{*3}\right]}^{1/2}}\;\text{and}\;\Phi_{s}=\frac{Q_{s}^{*}}{U_{0}^{*}D_{0}^{*}}.$$

In the above, $Q_{b}^{*}=(Q_{bx}^{*},Q_{by}^{*})$ is the bedload flux vector, $Q_{s}^{*}=(Q_{sx}^{*},Q_{sy}^{*})$ is the suspended load flux vector and $\rho_{0}$ is the porosity of particles. We consider the channel-banks to be impermeable to the flow and the sediment fluxes, so that the quantities $U_{y}$, $\Phi_{by}$ and $\Phi_{sy}$ vanish at the channel-banks (y=±1).

The bedload flux vector $\Phi_{b}$ is expressed as

3.8 $$\Phi_{b}=(\Phi_{bx},\Phi_{by})=(\cos\chi ,\sin\chi )\Phi ,$$

where χ is the angle that the resultant bedload flux makes with the longitudinal direction and Φ is the bedload flux intensity. The χ is expressed as follows [46,47]:

3.9 $$\chi =\sin^{-1}\left[\frac{U_{y}}{{\left(U_{x}^{2}+U_{y}^{2}\right)}^{1/2}}-\frac{\vartheta }{\beta \Theta^{1/2}}\cdot \frac{\partial R}{\partial y}\right],$$

where ϑ is the transverse slope coefficient.

For the bedload flux intensity Φ, we use Meyer-Peter & Müller’s formula as follows [48]:

3.10 $$\Phi =8{\left(\Theta -\Theta_{c}\right)}^{3/2}.$$

For the estimation of $\Theta_{c}$, we use the $\Theta_{c}(D_{*})$ relationships of Cao *et al.* [49].

Bolla Pittaluga & Seminara [37] presented an analytical solution for the estimation of suspended load flux in slowly varying flows by means of asymptotic expansion of the exact solution of the advection–diffusion equation of sediment concentration. In this study, to obtain the suspended load flux vector, we employ the two-dimensional extension of the depth-averaged formulation of Bolla Pittaluga & Seminara [37], as was done by Federici & Seminara [30] and Bertagni & Camporeale [32]. The perturbation approach assumes that both the advective and unsteady effects are trivial compared with the gravitational settling and turbulent diffusion. This suggests $\delta_{p}=[U_{0}^{*}D_{0}^{*}/(w_{s}^{*}\lambda^{*})]$ to be much smaller than unity. As the $\delta_{p}$ depends on the priori unknown wavelength of riverbed-patterns, we define another small parameter δ as $\delta =\delta_{p}\lambda^{*}/B^{*}=U_{0}^{*}/(\beta w_{s}^{*})$ [30,32].

The suspended load flux vector is expressed as follows [37]:

3.11 $$\Phi_{s}=(U_{x},U_{y})D\psi ,$$

where the function ψ is expanded as $\psi =\psi_{0}+\delta \psi_{1}+O(\delta^{2})$. Here, $\psi_{0}$ corresponds to the uniform flow condition and $\psi_{1}$ is the correction to ψ at O(δ). This correction takes into account the weak non-equilibrium effects due to the spatial change in the flow field. The $\psi_{0}$ and $\psi_{1}$ are expressed as follows [37]:

3.12 $$\psi_{0}=\frac{C_{0}K_{0}}{1-\rho_{0}},\;\psi_{1}=\frac{K_{1}D}{1-\rho_{0}}\left(U_{x}\frac{\partial C_{0}}{\partial x}+U_{y}\frac{\partial C_{0}}{\partial y}\right),$$

where $C_{0}$ is the depth-averaged suspended particle concentration at the leading order, and $K_{0}$ and $K_{1}$ are functional parameters.

The $C_{0}$ is expressed as follows [30]:

3.13 $$C_{0}=\frac{1}{1-a}C_{a}I_{1}\;\text{with}\;I_{1}=\int_{a}^{1}{\left(\frac{a}{1-a}\cdot \frac{1-z}{z}\right)}^{Z}\;\text{d}z.$$

In the above, $a=a^{*}/D^{*}$, $z=z^{*}/D^{*}$, $a^{*}$ is the reference level, $C_{a}$ is the reference concentration, Z is the Rouse number [$=w_{s}^{*}/(\kappa u_{f}^{*})$] and κ is the von Kármán coefficient (=0.41). In this study, we use van Rijn’s [44] empirical formulae to obtain a and $C_{a}$.

The $K_{0}$ in equation (3.12) is expressed as follows [30]:

3.14 $$K_{0}=\frac{C_{f}^{1/2}}{\kappa }(1-a)\left(\frac{I_{2}}{I_{1}}+K_{2}\right)\;\text{with}\;I_{2}=\int_{a}^{1}(\ln z+1.84z^{2}-1.56z^{3}){\left(\frac{a}{1-a}\cdot \frac{1-z}{z}\right)}^{Z}\;\text{d}z.$$

In the above, $K_{2}=0.777+\kappa C_{f}^{-1/2}$.

In addition, the $K_{1}$ in equation (3.12) is expressed as follows [30,37]:

3.15 $$K_{1}=\frac{C_{f}^{1/2}}{\kappa }\left[\int_{a}^{1}C_{12}(\ln z+1.84z^{2}-1.56z^{3})\;\text{d}z-\ln z_{0}\int_{a}^{1}C_{12}\text{d}z\right],$$

where $z_{0}=z_{0}^{*}/D^{*}$, $z_{0}$ is the zero-velocity level at which the time-averaged longitudinal velocity becomes zero (in accord with the classical logarithmic law) and $C_{12}$ is a function. The $C_{12}$ can be found by imposing the boundary conditions, namely $\partial C_{12}/\partial z=0$ at z=a and $C_{12}=0$ at z=1, to the following differential problem:

3.16 $$\frac{\partial C_{12}}{\partial z}+Z^{-1}\frac{\partial }{\partial z}\left[z(1-z)\frac{\partial }{\partial z}\right]C_{12}=\frac{(1-a)C_{f}^{1/2}}{\kappa I_{1}}\left(\ln\frac{z}{z_{0}}+1.84z^{2}-1.56z^{3}\right){\left(\frac{a}{1-a}\cdot \frac{1-z}{z}\right)}^{Z}.$$

## 4. Stability analysis

We expand the set of variables $V={\left(U_{x},U_{y},D,R\right)}^{⊺}$ as

4.1 $$V=V_{0}+\hat{V}={\left(1,0,1,R_{0}\right)}^{⊺}+{\left({\hat{U}}_{x},{\hat{U}}_{y},\hat{D},\hat{R}\right)}^{⊺},$$

where $R_{0}$ refers to the value of R at the undisturbed state. We express the components of bed shear stress and bedload flux vectors as

4.2 $$T_{x}=C_{f0}(1+c_{1}{\hat{U}}_{x}+c_{2}\hat{D}),\;T_{y}=C_{f0}{\hat{U}}_{y}$$

4.3 $$\begin{array}{rl} & \Phi_{bx}=\Phi_{0}(1+c_{3}{\hat{U}}_{x}+c_{4}\hat{D}),\\ & \Phi_{by}=\Phi_{0}\left[-{\hat{U}}_{x}{\hat{U}}_{y}+(1+c_{3}{\hat{U}}_{x}+c_{4}\hat{D})\left({\hat{U}}_{y}-\frac{\vartheta }{\beta \Theta_{0}^{1/2}}\cdot \frac{\partial \hat{R}}{\partial y}\right)+\frac{0.5\vartheta (c_{1}{\hat{U}}_{x}+c_{2}\hat{D})}{\beta \Theta_{0}^{1/2}}\cdot \frac{\partial \hat{R}}{\partial y}\right], \end{array}$$

where $C_{f0}$, $\Phi_{0}$ and $\Theta_{0}$ are the conductance factor, bedload flux intensity and Shields number, respectively, at the undisturbed state and the coefficients $c_{1-4}$ are given in appendix A. Further, in order to expand the suspended load flux vector, we express $\psi_{0}$ and $\psi_{1}$ as

4.4 $$\psi_{0}=\psi_{00}(1+c_{5}{\hat{U}}_{x}+c_{6}\hat{D}),\;\psi_{1}=c_{7}\frac{\partial {\hat{U}}_{x}}{\partial x}+c_{8}\frac{\partial \hat{D}}{\partial x},$$

where $\psi_{00}$ refers to the value of $\psi_{0}$ at the undisturbed state. The coefficients $c_{5-8}$ are given in appendix A.

Substituting equations (4.1)–(4.4) into equations (2.3)–(2.5) and (3.6), we obtain

4.5 $$L_{0}\frac{\partial \hat{V}}{\partial t}=L_{m}\hat{V}+\text{N}(\hat{V})+O({\hat{V}}^{3}),$$

where $L_{0}$ is the 4×4 matrix whose elements are zero except the lower right entry (being equal to unity), $L_{m}$ is the 4×4 matrix differential operator and the term $N(\hat{V})$ contains all the second-order nonlinearities. The perturbations can be expanded in terms of the eigenfunctions as follows [32,50]:

4.6 $$\hat{V}(x,y,t)=\sum_{\begin{array}{c} p=-\infty \\ p\neq 0 \end{array}}^{p=\infty }\sum_{m=1}^{n}A_{m,p}(t){\hat{v}}_{m}(pk,y)\text{e}\text{xp}(\text{i}pkx),$$

where $A_{m,p}$ corresponds to the amplitude of the longitudinal mode p and the transverse mode m, k is the dimensionless longitudinal wavenumber and i is the unit imaginary number. In the above expansion, the eigenfunctions are the product of eigenvectors ${\hat{v}}_{m}$ and the exponential term. We write the eigenvectors ${\hat{v}}_{m}$ as follows [32]:

4.7 $${{\hat{v}}_{m}|}_{m=\text{odd}}={\left[{\hat{u}}_{xm},{\hat{u}}_{ym}\cot\left(\frac{m\pi y}{2}\right),{\hat{d}}_{m},{\hat{r}}_{m}\right]}^{⊺}\sin\left(\frac{m\pi y}{2}\right)+\text{c.c.}$$

and

4.8 $${{\hat{v}}_{m}|}_{m=\text{even}}={\left[{\hat{u}}_{xm},{\hat{u}}_{ym}\tan\left(\frac{m\pi y}{2}\right),{\hat{d}}_{m},{\hat{r}}_{m}\right]}^{⊺}\cos\left(\frac{m\pi y}{2}\right)+\text{c.c.},$$

where c.c. stands for the complex conjugate. The eigenvectors ${\hat{v}}_{m}$ are the product of another vector ${\hat{u}}_{m}$ depending on the wavenumber of perturbation and the corresponding Fourier harmonics satisfying the boundary conditions. Note that the subscript m denotes the variable associated with the transverse mode.

### (a) Linear perspective

Linear analysis leads to the prediction of the favourable domain of the pattern formation. In the linear analysis, we consider only the fundamental longitudinal mode, p=1. Therefore, each transverse mode leads to an independent problem. The amplitude is considered to grow exponentially with time as $A_{m,p}\propto \exp(\omega_{m}t)$, where $\omega_{m}$ is the complex number, whose real and imaginary parts represent the growth rate and the dimensionless frequency, respectively. With p=1, equations (4.6)–(4.8) are substituted into equation (4.5) and the nonlinearities are ignored. The resultant linear system is

4.9 $$(L_{0}\omega_{m}-L_{m}){\hat{u}}_{m}=0,$$

where elements $l_{ij}$ of the matrix $L_{m}$ are expressed as

4.10 $$\begin{array}{rl} & l_{11}=-(\text{i}k+\beta C_{f0}c_{1}),\;l_{12}=l_{21}=l_{34}=0,\;l_{13}=-(c_{2}-1)\beta C_{f0}-\frac{\text{i}k}{Fr^{2}},\;l_{14}=-\frac{\text{i}k}{Fr^{2}},\\ & l_{22}=-(\text{i}k+\beta C_{f0}),\;l_{23}=l_{24}={\left(-1\right)}^{m}\frac{\pi m}{2Fr^{2}},\;l_{31}=l_{33}=-\text{i}k,\;l_{32}={\left(-1\right)}^{m+1}\frac{\pi m}{2Fr^{2}},\\ & l_{41}=k^{2}\delta c_{7}-\text{i}kc_{3}\Phi_{0}Q_{0}-\text{i}k(1+c_{5})\psi_{00},\;l_{42}={\left(-1\right)}^{m+1}\frac{\pi m}{2}(\Phi_{0}Q_{0}+\psi_{00}),\\ & l_{43}=k^{2}\delta c_{8}-\text{i}kc_{4}\Phi_{0}Q_{0}-\text{i}k(1+c_{6})\psi_{00},\;l_{44}=-\frac{m^{2}\pi^{2}\Phi_{0}Q_{0}\vartheta }{4\beta \Theta^{1/2}}. \end{array}$$

The solvability condition of equation (4.9) leads to the dispersion relationship from which the eigenvalue $\omega_{m}$ can be obtained. The dispersion relationship is expressed as

4.11 $$\begin{array}{rl} & \left(l_{44}-\omega_{m})(l_{13}l_{22}l_{31}-l_{11}l_{22}l_{33}+l_{11}l_{23}l_{32})=l_{11}l_{24}(l_{32}l_{43}-l_{33}l_{42}\right)\\ & \;+l_{13}l_{24}(l_{31}l_{42}-l_{32}l_{41})+l_{14}l_{22}(l_{31}l_{43}-l_{33}l_{41})-l_{14}l_{23}(l_{31}l_{42}-l_{32}l_{41}). \end{array}$$

### (b) Weakly nonlinear perspective

An analytical solution for the finite amplitude of mega riverbed-patterns can be obtained by performing a stability analysis from the weakly nonlinear perspective. Although the multiple-scale technique has been used to carry out the weakly nonlinear analysis [3], the finite amplitude solutions obtained from this technique are limited to the surroundings of the critical point. Bertagni & Camporeale [32,51] used the centre-manifold-projection technique to obtain an analytical solution for the finite amplitude of morphodynamic patterns. Following the procedure of Bertagni & Camporeale [32], this analysis aims at obtaining the finite amplitude solution for the fundamental mode (m=1).

Initially, we solve the following linear adjoint eigenvalue problem:

4.12 $$(L_{0}^{†}{\bar{\omega }}_{m}-L_{m}^{†}){\hat{u}}_{m}^{†}=0,$$

where the superscript † stands for the adjoint operator and the overbar denotes the complex conjugate. In accordance with the boundary conditions, the inner product defining the adjoint operator is expressed as

4.13 $$\int_{-1}^{1}(La_{1})\cdot {\bar{a}}_{2}\text{d}y=\int_{-1}^{1}a_{1}\cdot (\bar{L^{†}a_{2}})\;\text{d}y,$$

where L is the linear operator, and $a_{1}$ and $a_{2}$ are the generic vectors. In this study, $L_{0}=L_{0}^{†}$ and $L_{m}^{†}$ represents the conjugate transpose of $L_{m}$. It is worth mentioning that for $L=L_{0}$, the eigenvectors ${\hat{v}}_{m}$ and adjoint eigenvectors ${\hat{v}}_{m}^{†}$ become orthonormal after the proper normalization. Hence, we write

4.14 $$\int_{-1}^{1}(L_{0}{\hat{v}}_{i})\cdot \bar{{\hat{v}}_{j}^{†}}\text{d}y=\delta_{ij},$$

where $\delta_{ij}$ is the Kronecker delta function, and i and j are the transverse modes. Unlike the linear analysis, the nonlinear interactions of different modes are to be accounted for in the weakly nonlinear analysis. In this study, we consider that the contributions from the higher-order modes are negligible. Therefore, the expansion of equation (4.6) is considered up to p=2 and m=2.

We substitute the expansion of equation (4.6) into equation (4.5), take the inner product of the resulting equation with the adjoint eigenfunctions and subsequently collect the terms of the same Fourier modes. This operation yields the amplitude equation of the fundamental and two other stable modes as follows:

4.15 $$\frac{\text{d}A_{1,1}}{\text{d}t}=\omega_{1}(k)A_{1,1}+G_{1}{\bar{A}}_{1,1}A_{1,2}+G_{2}{\bar{A}}_{1,1}A_{2,2}$$

and

4.16 $${\frac{\text{d}A_{m,2}}{\text{d}t}|}_{m=1,2}=\omega_{m}(2k)A_{m,2}+H_{m}A_{1,1}^{2}+\ldots ,$$

where ${\bar{A}}_{11}$ stands for $A_{1,-1}$, and $G_{m}$ and $H_{m}$ (for m = 1 and 2) are the nonlinear interaction coefficients. The $G_{m}$ and $H_{m}$ can be expressed as follows [50]:

4.17 $$G_{m}=2\int_{-1}^{1}\text{N}[{\hat{v}}_{1}(-k,y)\exp(-\text{i}kx),{\hat{v}}_{m}(2k,y)\exp(2\text{i}kx)]\cdot \bar{{\hat{v}}_{1}^{†}}(k,y)\exp(-\text{i}kx)\text{d}y$$

and

4.18 $$H_{m}=\int_{-1}^{1}\text{N}[{\hat{v}}_{1}(k,y)\exp(\text{i}kx),{\hat{v}}_{1}(k,y)\exp(\text{i}kx)]\cdot \bar{{\hat{v}}_{m}^{†}}(2k,y)\exp(-2\text{i}kx)\text{d}y.$$

The centre-manifold-projection technique facilitates to project the amplitudes of the stable modes on the unstable ones. Therefore, the stable amplitudes $A_{m,2}$ can be written as a nonlinear combination of $A_{1,1}$ and ${\bar{A}}_{1,1}$ as

4.19 $$A_{m,2}=p_{1}A_{1,1}^{2}+p_{2}A_{1,1}{\bar{A}}_{1,1}+p_{3}{\bar{A}}_{1,1}^{2}+O(A_{1,1}^{3}),$$

where $p_{1}$, $p_{2}$ and $p_{3}$ are the projection coefficients. After neglecting the higher-order terms, the time derivative of equation (4.19) is expressed as

4.20 $$\frac{\text{d}A_{m,2}}{\text{d}t}=2p_{1}A_{1,1}\frac{\text{d}A_{1,1}}{\text{d}t}+p_{2}\left({\bar{A}}_{1,1}\frac{\text{d}A_{1,1}}{\text{d}t}+A_{1,1}\frac{\text{d}{\bar{A}}_{1,1}}{\text{d}t}\right)+2p_{3}{\bar{A}}_{1,1}\frac{\text{d}{\bar{A}}_{1,1}}{\text{d}t}.$$

Substituting equations (4.15), (4.16) and (4.19) into equation (4.20), we obtain

4.21 $$\begin{array}{rl} & [p_{1}\omega_{m}(2k)-2p_{1}\omega_{1}(k)+H_{m}]A_{1,1}^{2}+p_{2}[\omega_{m}(2k)-\omega_{1}(k)-{\bar{\omega }}_{1}(k)]A_{1,1}{\bar{A}}_{1,1}\\ & \;+p_{3}[\omega_{m}(2k)-2{\bar{\omega }}_{1}(k)]{\bar{A}}_{1,1}^{2}=0. \end{array}$$

To solve the above equation, we consider $p_{2}=p_{3}=0$. Therefore, the coefficient $p_{1}$ is expressed as

4.22 $$p_{1}=-\frac{H_{m}}{\omega_{m}(2k)-2\omega_{1}(k)}.$$

In addition, the amplitudes of the stable modes can be expressed as

4.23 $$A_{m,2}=-\frac{H_{m}}{\omega_{m}(2k)-2\omega_{1}(k)}A_{1,1}^{2}.$$

Substituting equation (4.23) into equation (4.15), the Stuart–Landau equation describing the amplitude dynamics of the fundamental mode can be obtained as

4.24 $$\frac{\text{d}A_{1,1}}{\text{d}t}=[\omega_{1}(k)-\sigma {|A_{1,1}|}^{2}]A_{1,1},$$

where the coefficient σ is expressed as

4.25 $$\sigma =\frac{G_{1}H_{1}}{\omega_{1}(2k)-2\omega_{1}(k)}+\frac{G_{2}H_{2}}{\omega_{2}(2k)-2\omega_{1}(k)}.$$

The equilibrium amplitude of the fundamental mode, e.g. alternate bars, can be obtained by setting the time derivative in equation (4.24) equal to zero. Therefore, the dimensionless amplitude of alternate bars can be expressed as

4.26 A=2[Re(ω1(k)σ)]1/2.

In the above, the factor 2 is involved due to the complex conjugation.

## 5. Results and discussion

In the numerical computations, we consider the mass density of fluid $\rho_{f}=1000\;{\text{kg m}}^{-3}$, mass density of particles $\rho_{p}=2650\;{\text{kg m}}^{-3}$, porosity of particles $\rho_{0}$ = 0.3, transverse slope coefficient ϑ=0.56 and acceleration due to gravity $g=9.81\;{\text{m s}}^{-2}$.

**Figure 2.**
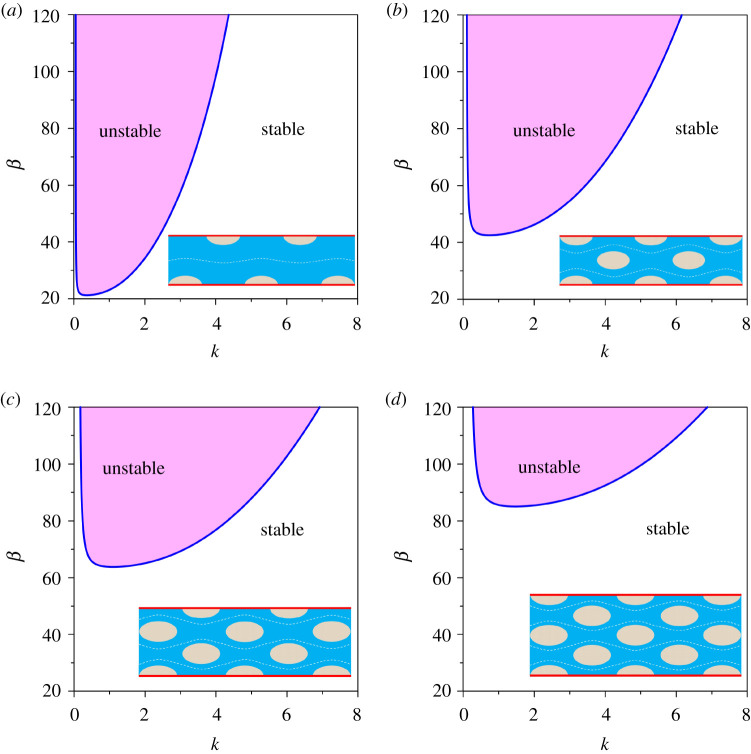
Aspect ratio β versus dimensionless wavenumber k in a transitional flow regime ($R_{*}=50$) for different transverse modes m: (*a*) m=1, (*b*) m=2, (*c*) m=3 and (*d*) m=4. (Online version in colour.)

To present the results from the linear perspective, we first examine how the mega riverbed-patterns evolve with different transverse modes. To this end, we consider a transitional flow regime and set the characteristic value of the shear Reynolds number as $R_{*}=50$. In addition, the reference values of Shields number and the relative roughness number are taken as Θ=0.2 and $d_{r}=0.0001$, respectively. This combination produces the flow Froude number Fr=0.166. Figure 2 shows the aspect ratio β as a function of dimensionless wavenumber k for different transverse modes m (=1,2,3 and 4). Note that each transverse mode corresponds to the specific rhythmic patterns in both longitudinal and transverse directions, as conceptually sketched in the insets of figure 2*a*–*d*. To be specific, m=1 corresponds to one row of alternate bars, while m=2 corresponds to two rows of alternate bars, i.e. central bars. In addition, m=3 and 4 comprising three and four rows of alternate bars correspond to the channelized bars. In each panel of figure 2, the stability map for a given m is shown on the β(k) plane. The marginal stability curve (solid line) makes a distinction between the unstable zone (shaded portion) and the stable zone (white portion). It defines the locus of the vanishing growth rate on the β(k) plane, i.e. $\text{Re}(\omega_{m})=0$. Therefore, the unstable and stable zones correspond to $\text{Re}(\omega_{m})>0$ and $\text{Re}(\omega_{m})<0$, respectively. From a physical viewpoint, in the unstable zone (zone confined to the marginal stability curve), the incipient riverbed-patterns with a given transverse mode grow with time. On the other hand, in the stable zone (zone outside the marginal stability curve), the incipient riverbed-patterns with a given transverse mode decay yielding no patterns. Figure 2 shows that the patterns are unable to grow below a threshold aspect ratio $\beta_{c}$ for which the slope of the β(k) curve disappears, i.e. dβ/dk=0. The $\beta_{c}$ therefore sets the limit for a stable alluvial channel having no riverbed-patterns. This indicates that the patterns are likely to form if the aspect ratio is larger than its threshold value. It is evident that the threshold aspect ratio increases with an increase in transverse mode (figure 2). For a given $\beta \gg \beta_{c}$, the unstable zone captures longer wavenumbers as the transverse mode increases. Note that in the subsequent sections, we consider only the fundamental mode (m=1).

**Figure 3.**
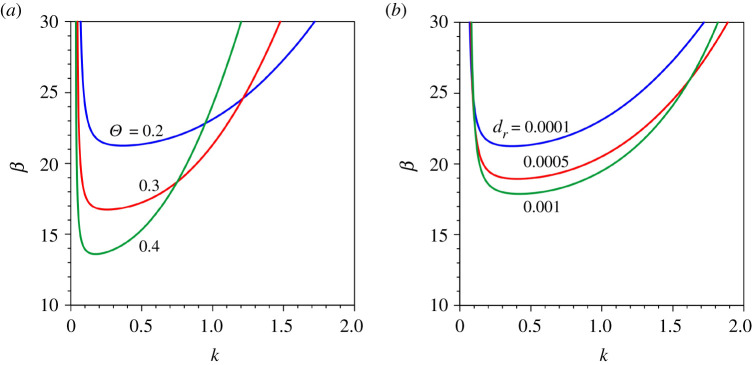
Aspect ratio β versus dimensionless wavenumber k in a transitional flow regime ($R_{*}=50$) for the fundamental mode (m=1): (*a*) Θ=0.2, 0.3 and 0.4 and (*b*) $d_{r}=0.0001$, 0.0005 and 0.001. (Online version in colour.)

Figure 2 does not offer an understanding of how the marginal stability curve for a given set ($R_{*},m$) is sensitive to the Shields number and the relative roughness number. To explore this aspect, figure 3 shows the marginal stability curves for different Shields numbers and relative roughness numbers. The shear Reynolds number and the transverse mode are considered to be $R_{*}=50$ and m=1 (fundamental mode), respectively. In figure 3*a*, the β(k) curves are shown for a given relative roughness number ($d_{r}=0.0001$) and different Shields numbers (Θ=0.2, 0.3 and 0.4 yielding different flow Froude numbers Fr=0.166, 0.204 and 0.235, respectively). As the shear Reynolds number is considered to be constant ($R_{*}=50$), an increase in Shields number in figure 3*a* refers to a reduction in particle size, as evident from equation (3.4). The unstable zone for a given Shields number increases as the Shields number increases (figure 3*a*). This is credited to the presence of significant sediment suspension that causes a destabilizing effect. The threshold aspect ratio reduces with an increase in Shields number. However, the longer wavenumbers at a large aspect ratio are stabilized as the Shields number increases. On the other hand, in figure 3*b*, the β(k) curves are shown for a given Shields number (Θ=0.2) and different relative roughness numbers ($d_{r}=0.0001$, 0.0005 and 0.001 yielding different flow Froude numbers Fr=0.166, 0.318 and 0.416, respectively). As the shear Reynolds number and the Shields number are kept constant ($R_{*}=50$ and Θ=0.2) in figure 3*b*, the particle size becomes a constant. It follows that an increase in relative roughness number produces a reduction in the flow depth. This causes an increase in the friction factor. The unstable zone for a given relative roughness number increases as the relative roughness number increases (figure 3*b*), lowering the threshold aspect ratio. This causes the threshold aspect ratio to reduce.

**Figure 4.**
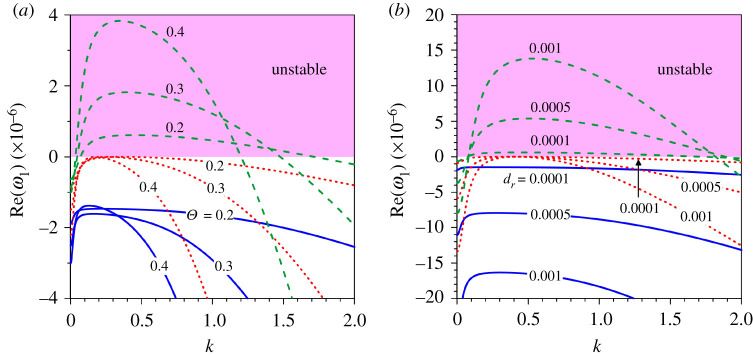
Dimensionless growth rate $\text{Re}(\omega_{1})$ versus dimensionless wavenumber k in a transitional flow regime ($R_{*}=50$) for the fundamental mode (m=1) corresponding to three distinct conditions, $\beta <\beta_{c}$ (blue solid lines), $\beta =\beta_{c}$(red dotted lines) and $\beta >\beta_{c}$ (green dashed lines): (*a*) Θ=0.2, 0.3 and 0.4 and (*b*) $d_{r}=0.0001$, 0.0005 and 0.001. (Online version in colour.)

It is interesting to figure out how the growth rate of patterns evolves with the Shields number and the relative roughness number. To this end, we consider the fundamental mode (m=1) and set the shear Reynolds number as $R_{*}=50$ (transitional flow regime). Figure 4*a*,*b* displays the dimensionless growth rate $\text{Re}(\omega_{1})$ as a function of dimensionless wavenumber k for different Shields numbers and relative roughness numbers, respectively. In each panel, the growth rate is examined for three different cases, $\beta <\beta_{c}$, $\beta =\beta_{c}$ and $\beta >\beta_{c}$. In figure 4*a*, the $\text{Re}(\omega_{1})(k)$ curves are shown for a given relative roughness number ($d_{r}=0.0001$) and different Shields numbers (Θ=0.2, 0.3 and 0.4 yielding different flow Froude numbers Fr=0.166, 0.204 and 0.235, respectively). The growth rate curves corresponding to $\beta <\beta_{c}$, $\beta =\beta_{c}$ and $\beta >\beta_{c}$ are shown by solid, dotted and dashed lines, respectively. As the $\text{Re}(\omega_{1})=0$ sets the limit for the stable patterns, the shaded zone above $\text{Re}(\omega_{1})=0$ represents the unstable zone. For $\beta <\beta_{c}$ (solid lines), the growth rate is expected to be negative. It is apparent that the growth rate for a given Shields number increases (decrease in negative magnitude) with a marginal increase in wavenumber depicting a peak at a shorter wavenumber, and thereafter it reduces (increase in negative magnitude) with a further increase in wavenumber (figure 4*a*). In general, the growth rate for a longer wavenumber dampens (increase in negative magnitude) as the Shields number increases. For $\beta =\beta_{c}$ (dotted lines), the growth rate for a given Shields number follows a similar trend with the wavenumber. Note that the growth rate for a given Shields number is negative except at the critical wavenumber, where it is zero. The growth rate for a longer wavenumber diminishes (increase in negative magnitude) as the Shields number increases. For $\beta >\beta_{c}$ (dashed lines), the growth rate for a given Shields number changes from negative to positive at a shorter wavenumber, attaining a positive peak with an increase in wavenumber and then it reduces with a further increase in wavenumber. The growth rate makes another changeover from positive to negative at a longer wavenumber and reduces (increase in negative magnitude) as the wavenumber increases (figure 4*a*). Importantly, for a given Shields number and $\beta >\beta_{c}$, the crossings (where the growth rate changes its sign) correspond to the two points on the marginal stability curve β(k). For a given intermediate wavenumber, the growth rate enhances with the Shields number. However, for a given shorter (or longer) wavenumber, it reduces (increase in negative magnitude) as the Shields number increases. In figure 4*b*, the $\text{Re}(\omega_{1})(k)$ curves are shown for a given Shields number (Θ=0.2) and different relative roughness numbers ($d_{r}$ = 0.0001, 0.0005 and 0.001 yielding different flow Froude numbers Fr=0.166, 0.318 and 0.416, respectively). For $\beta <\beta_{c}$ (solid lines), the growth rate for a given relative roughness number increases (decrease in negative magnitude) with a small increase in wavenumber attaining a peak and thereafter reduces (increase in negative magnitude) with a further increase in wavenumber. The growth rate for a given wavenumber diminishes (increase in negative magnitude) with an increase in relative roughness. For $\beta =\beta_{c}$ (dotted lines), the growth rate for a given relative roughness number is negative everywhere except at the critical wavenumber, which produces a vanishing growth rate. The growth rate for a given wavenumber reduces (increase in negative magnitude) as the relative roughness number increases. Similar to figure 4*a*, for $\beta >\beta_{c}$ (dashed lines), the growth rate for a given relative roughness number changes its sign twice at shorter and longer wavenumbers (figure 4*b*). For a given intermediate wavenumber, the growth rate increases with an increase in relative roughness number. However, for a given shorter (or longer) wavenumber it decreases (increase in negative magnitude) as the relative roughness number increases.

**Figure 5.**
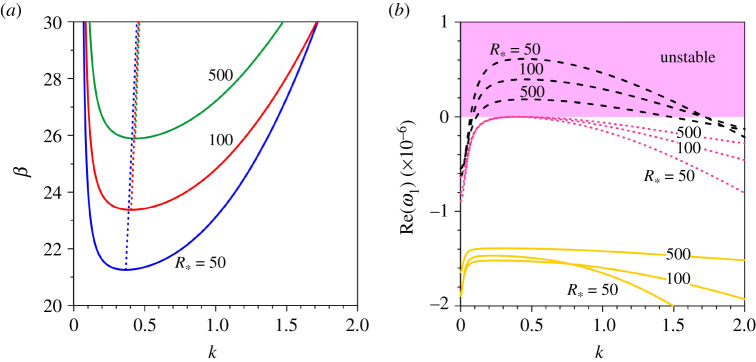
(*a*) Aspect ratio β versus dimensionless wavenumber k for different shear Reynolds numbers ($R_{*}=50$, 100 and 500) shown by blue, red and green solid lines,respectively (dotted lines show the loci of the maximum growth rate) and (*b*) dimensionless growth rate $\text{Re}(\omega_{1})$ versus dimensionless wavenumber k for different shear Reynolds numbers ($R_{*}=50$, 100 and 500) corresponding to three distinct conditions, $\beta <\beta_{c}$ (yellow solid lines), $\beta =\beta_{c}$ (pink dotted lines) and $\beta >\beta_{c}$ (black dashed lines). (Online version in colour.)

Figures 3 and 4 highlight the marginal stability curves and the growth rates in a transitional flow regime. However, riverbed-patterns are sensitive to flow regimes. Therefore, the evolution of patterns with shear Reynolds number is an important aspect. Figure 5*a* shows the marginal stability curves corresponding to the fundamental mode (m=1) for different shear Reynolds numbers ($R_{*}=50$, 100 and 500). The Shields number and the relative roughness number are taken as Θ=0.2 and $d_{r}=0.0001$, respectively. This combination produces different but nearly equal flow Froude numbers Fr=0.166, 0.168 and 0.169. Note that $R_{*}=100$ and 500 correspond to a rough flow regime. As the Shields number is kept constant (Θ=0.2), an increase in shear Reynolds number refers to an increase in particle size (see equation (3.4)). The unstable zone for a given shear Reynolds number reduces with an increase in shear Reynolds number due to an increase in particle size (figure 5*a*). Therefore, the threshold aspect ratio increases as the shear Reynolds number increases. The longer wavenumbers at a large aspect ratio are stabilized as the shear Reynolds number increases. It is worth emphasizing that for a given shear Reynolds number $R_{*}$, the growth rate of patterns for a given aspect ratio $\beta (>\beta_{c})$ attains a maximum value at some characteristic wavenumber, called the maximum unstable wavenumber. For a given shear Reynolds number, the locus of the maximum growth rate is shown in figure 5*a* (see dotted lines). In fact, the loci of the maximum growth rate for different shear Reynolds numbers almost coincide. Figure 5*b* shows the dimensionless growth rate $\text{Re}(\omega_{1})$ as a function of wavenumber k corresponding to $\beta <\beta_{c}$, $\beta =\beta_{c}$ and $\beta >\beta_{c}$ for different shear Reynolds numbers ($R_{*}=50$, 100 and 500). For $\beta <\beta_{c}$ (solid lines), the growth rate for a given shear Reynolds number increases (decrease in negative magnitude) with a small increase in wavenumber reaching a peak and thereafter decreases (increase in negative magnitude) with a further increase in wavenumber. The growth rate for a given longer wavenumber increases (decrease in negative magnitude) as the shear Reynolds number increases. For $\beta =\beta_{c}$ (dotted lines), the growth rate for a given longer wavenumber also increases (decrease in negative magnitude) as the shear Reynolds number increases. However, for $\beta >\beta_{c}$ (dashed lines), the growth rate for a given intermediate wavenumber lessens as the shear Reynolds number increases. Note that the growth rate for a given shear Reynolds number changes its sign twice at shorter and longer wavenumbers (figure 5*b*).

**Figure 6.**
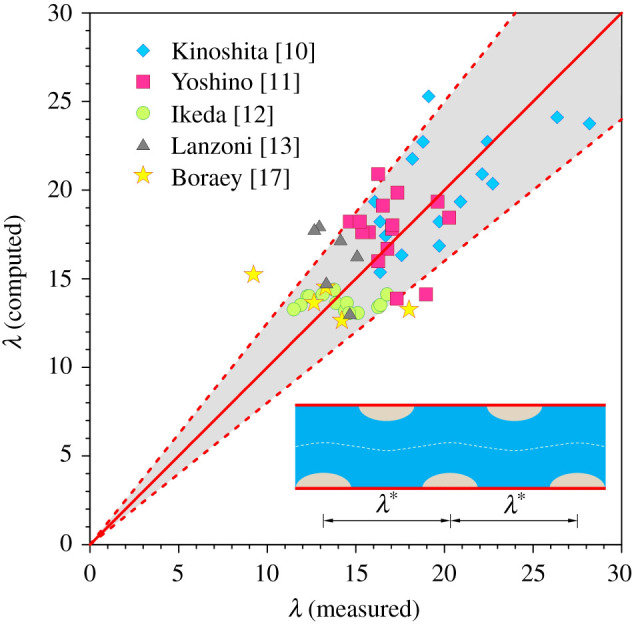
Comparison of the theoretical predictions of dimensionless wavelength of patterns with the experimental data. The solid line represents the line of perfect agreement. The dashed lines bound the ±20% error band. (Online version in colour.)

Figure 6 offers comparison of the computed dimensionless wavelength of patterns with the available experimental data [10–13,17]. The comparison is performed for the fundamental mode of riverbed-patterns, e.g. alternate bars (see the conceptual sketch in the inset of figure 6) by making the pertinent variables on a par with the experimental data. A brief summary of the experimental conditions used for the comparison is given in table 1. Note that some of the experimental data in figure 6 primarily correspond to the bedload-dominated channels. Therefore, the theoretical results for them in figure 6 are obtained by neglecting the role of sediment suspension. In this context, it is worth mentioning that an accurate estimation of the transverse slope coefficient ϑ from the experimental data is a difficult proposition. Therefore, the ϑ is kept as a free parameter. It is found that for ϑ≈0.2, the theoretical predictions are in satisfactory agreement with the experimental data, as most of the data are confined to the ±20% error band (figure 6). In fact, the choice of ϑ=0.2 is close to the previously proposed value (=0.56) of the transverse slope coefficient [52].

**Table 1.** Brief summary of the experimental conditions.

author	Fr	Θ	$d_{r}$	$R_{*}$
Kinoshita [10]	1.04–1.98	0.064–0.311	0.069–0.266	24.6–374.1
Yoshino [11]	0.74–1.5	0.065–0.463	0.021–0.186	29.6–762.3
Ikeda [12]	0.77–1.16	0.062–0.098	0.036–0.133	112.4–147.3
Lanzoni [13]	0.32–0.69	0.147–0.366	0.006–0.014	40.7–64.3
Boraey [17]	0.67–1.33	0.088–0.102	0.026–0.107	108.7–116.8

**Figure 7.**
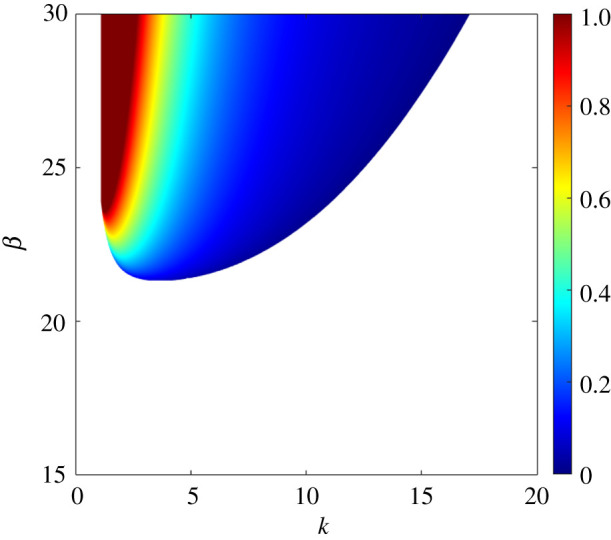
Contours of dimensionless amplitude A in a transitional flow regime ($R_{*}=50$) on the β(k) plane. (Online version in colour.)

To present the results from the weakly nonlinear perspective, we first explore the amplitude of patterns for a given set ($\beta ,k,R_{*},\Theta ,d_{r}$). Figure 7 shows the contours of dimensionless amplitude on the β(k) plane. The flow regime is considered to be transitional ($R_{*}=50$). The Shields number and the relative roughness number are taken as Θ=0.2 and $d_{r}=0.0001$, respectively. For a given aspect ratio $\beta (>\beta_{c})$, the amplitude varies with the wavenumber over a certain range. For a given aspect ratio, the amplitude amplifies towards the shorter wavenumbers. On the other hand, for a given wavenumber, the amplitude varies insignificantly with the aspect ratio (figure 7).

**Figure 8.**
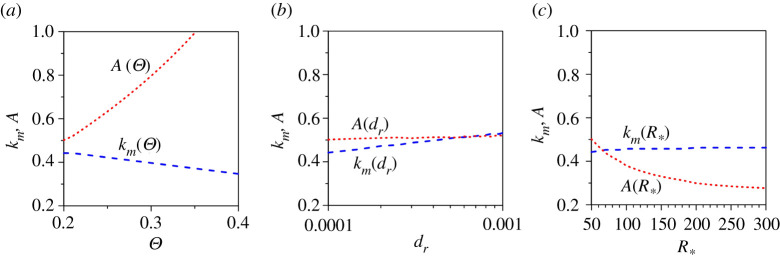
(*a*) Dimensionless maximum unstable wavenumber $k_{m}$ and dimensionless amplitude A versus Shields number Θ, (*b*) dimensionless maximum unstable wavenumber $k_{m}$ and dimensionless amplitude A versus relative roughness number $d_{r}$ and (*c*) dimensionless maximum unstable wavenumber $k_{m}$ and dimensionless amplitude A versus shear Reynolds number $R_{*}$. (Online version in colour.)

The sensitivity of the amplitude of patterns to the key variables remains a key aspect. Therefore, it is further interesting to envision how the amplitude of patterns varies with the pertinent variables, such as Θ, $d_{r}$ and $R_{*}$. Figure 8*a*–*c* shows the maximum unstable wavenumber $k_{m}$ and the dimensionless amplitude A as a function of the Shields number Θ, relative roughness number $d_{r}$ and shear Reynolds number $R_{*}$, respectively. In figure 8*a*, the $k_{m}(\Theta )$ and A(Θ) curves are shown for $d_{r}=0.0001$ and $R_{*}=50$. The maximum unstable wavenumber reduces with an increase in Shields number, while the amplitude increases. In figure 8*b*, the $k_{m}(d_{r})$ and $A(d_{r})$ curves are shown for Θ=0.2 and $R_{*}=50$ on a semi-logarithmic scale. The maximum unstable wavenumber increases marginally as the relative roughness number increases, while the amplitude remains nearly invariant with the relative roughness number. In addition, in figure 8*c*, the $k_{m}(R_{*})$ and $A(R_{*})$ curves are shown for Θ=0.2 and $d_{r}=0.0001$. The maximum unstable wavenumber appears to remain constant with an increase in shear Reynolds number, while the amplitude reduces attaining a constant value for large shear Reynolds numbers ($R_{*}>300$).

**Figure 9.**
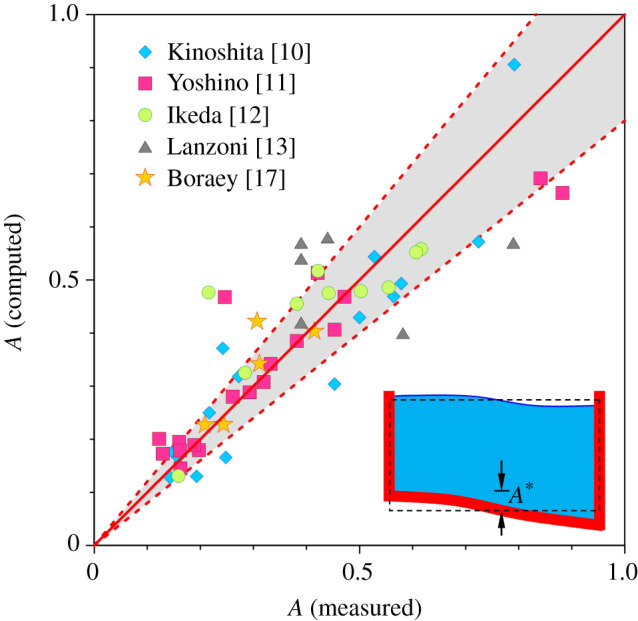
Comparison of the theoretical predictions of dimensionless amplitude of patterns with the experimental data. The solid line represents the line of perfect agreement. The dashed lines bound the ±20% error band. (Online version in colour.)

Figure 9 shows comparison of the computed dimensionless amplitude of patterns with the experimental data corresponding to the bedload-dominated channels [10–13,17]. The comparison is performed for the fundamental mode of riverbed-patterns, e.g. alternate bars (see the conceptual sketch in the inset of figure 9). The experimental conditions used for the comparison are furnished in table 1. The ϑ is set equal to 0.2, as was also considered in figure 6. The theoretical predictions match well with the experimental data, as most of the data excepting a few fall within the ±20% error band (figure 9).

## 6. Conclusion

We analyse the sensitivity of mega riverbed-patterns of finite amplitude without an external forcing from both linear and weakly nonlinear perspectives. The depth-averaged flow model and the particle transport model are coupled by means of the Exner equation, which governs the dynamics of the physical system that describes a steady-uniform flow in a straight channel with an erodible channel-bed. The stability analyses include the standard linearization technique and the centre-manifold-projection technique from linear and weakly nonlinear perspectives, respectively. The stability of mega riverbed-patterns is found to be largely sensitive to the channel aspect ratio, wavenumber of patterns, shear Reynolds number, Shields number and relative roughness number. The marginal stability, growth rate and amplitude of patterns are explored.

The unstable zone for a given Shields number increases with an increase in Shields number due to the destabilizing effect caused by the sediment suspension. In addition, the unstable zone for a given relative roughness number increases, as the relative roughness number increases. However, the unstable zone for a given shear Reynolds number reduces as the shear Reynolds number increases. For a given shear Reynolds number, Shields number and relative roughness number, the threshold aspect ratio increases with an increase in transverse mode.

Within the unstable zone, the growth rate for a given intermediate wavenumber increases with an increase in Shields number. Further, the growth rate for a given intermediate wavenumber increases as the relative roughness number increases. However, the growth rate for a given intermediate wavenumber reduces as the shear Reynolds number increases. The loci of the maximum growth rate for different shear Reynolds numbers approximately coincide.

Within the unstable zone, the amplitude of patterns intensifies towards shorter wavenumbers. The amplitude increases with an increase in Shields number, while it remains nearly constant with an increase in relative roughness number. However, the amplitude reduces as the shear Reynolds number increases.

From the linear perspective, this study reveals that among the chosen range of characteristic parameters, the marginal stability curve is more sensitive to the Shields number and the shear Reynolds number than to the relative roughness number. However, the growth rate is largely sensitive to the relative roughness number. From the weakly nonlinear perspective, the finite amplitude of patterns is most sensitive to the Shields number.

It is worth emphasizing that the limitation of the linear stability analysis is its inefficacy to predict the finite amplitude of patterns. This can be resolved by performing a weakly nonlinear stability analysis. However, the weakly nonlinear stability analysis is not capable of addressing the complete nonlinear interactions of the physical system. This may require a sophisticated numerical treatment.

Essentially, this study provides an insight into the stability of mega riverbed-patterns from both linear and weakly nonlinear perspectives. However, the theoretical results in the presence of sediment suspension are to be extensively validated with the experimental data, as most of the laboratory experiments were limited to the bedload-dominated channels. This aspect demands high-fidelity data of the wavelength and the amplitude of patterns forming in a laboratory environment, where suspended particles play a major role.

## Data accessibility

The paper does not have any additional data. The code for the computations is available at: https://drive.google.com/file/d/1TvUWc9U6fkExDsKT9EzHixDacing7Do-/view?usp=sharing.

## Appendix A. Coefficients $c_{1-8}$

The coefficients $c_{1-8}$ are expressed as

A 1 $$\begin{array}{rl} & c_{1}=2{\left(1-\frac{\Theta_{0}}{C_{f0}}\cdot \frac{\partial C_{f}}{\partial \Theta }\right)}^{-1},\;c_{2}=\frac{1}{C_{f0}}\cdot \frac{\partial C_{f}}{\partial D}{\left(1-\frac{\Theta_{0}}{C_{f0}}\cdot \frac{\partial C_{f}}{\partial \Theta }\right)}^{-1},\\ & c_{3}=\frac{\Theta_{0}}{\Phi_{0}}\cdot \frac{\partial \Phi }{\partial \Theta }c_{1},\;c_{4}=\frac{\Theta_{0}}{\Phi_{0}}\cdot \frac{\partial \Phi }{\partial \Theta }c_{2}+\frac{1}{\Phi_{0}}\cdot \frac{\partial \Phi }{\partial D},\\ & c_{5}=c_{1}\left\{\frac{\Theta_{0}}{2C_{f0}}\cdot \frac{\partial C_{f}}{\partial \Theta }+\frac{1.5\Theta_{0}}{\Theta_{0}-\Theta_{c}}-0.5{\left(I_{20}+K_{20}I_{10}\right)}^{-1}\left[Z_{0}(I_{21}+K_{20}I_{11})+\frac{\kappa \Theta_{0}I_{10}}{C_{f0}^{3/2}}\cdot \frac{\partial C_{f}}{\partial \Theta }\right]\right\},\\ & c_{6}=c_{2}\left\{\frac{1}{2}-\frac{1}{c_{2}}+\frac{1.5\Theta_{0}}{\Theta_{0}-\Theta_{c}}-0.5{\left(I_{20}+K_{20}I_{10}\right)}^{-1}\left[Z_{0}(I_{21}+K_{20}I_{11})+\frac{\kappa I_{10}}{C_{f0}^{1/2}}\right]\right\},\\ & c_{7}=\frac{c_{1}C_{a0}K_{10}}{\left(1-a)(1-\rho_{0}\right)}\left(\frac{1.5\Theta_{0}}{\Theta_{0}-\Theta_{c}}I_{10}-0.5Z_{0}I_{11}\right),\\ & c_{8}=\frac{c_{2}C_{a0}K_{10}}{\left(1-a)(1-\rho_{0}\right)}\left[\left(\frac{1.5\Theta_{0}}{\Theta_{0}-\Theta_{c}}-\frac{1}{c_{2}}\right)I_{10}-0.5Z_{0}I_{11}\right], \end{array}$$

where $Z_{0}$, $C_{a0}$, $I_{10}$, $I_{20}$ and $K_{20}$ are the Rouse number, reference concentration, $I_{1}$, $I_{2}$ and $K_{2}$, respectively, at the undisturbed state.

In the above equation, the integral functions $I_{11}$ and $I_{21}$ are expressed as

A 2 $$I_{11}=\int_{a}^{1}{\left(\frac{a}{1-a}\cdot \frac{1-z}{z}\right)}^{Z_{0}}\text{ln}\left(\frac{a}{1-a}\cdot \frac{1-z}{z}\right)\text{d}z$$

and

A 3 $$I_{21}=\int_{a}^{1}(\ln z+1.84z^{2}-1.56z^{3}){\left(\frac{a}{1-a}\cdot \frac{1-z}{z}\right)}^{Z_{0}}\text{ln}\left(\frac{a}{1-a}\cdot \frac{1-z}{z}\right)\text{d}z.$$
